# Application of Transgenic Zebrafish Models for Studying the Effects of Estrogenic Endocrine Disrupting Chemicals on Embryonic Brain Development

**DOI:** 10.3389/fphar.2022.718072

**Published:** 2022-02-11

**Authors:** Aya Takesono, Tetsuhiro Kudoh, Charles R. Tyler

**Affiliations:** Biosciences, College of Life and Environmental Sciences, University of Exeter, Exeter, United Kingdom

**Keywords:** estrogenic EDCs, transgenic zebrafish, estrogen biosensors, developmental neurotoxicity, risk assessment, estrogens

## Abstract

Endocrine disrupting chemicals (EDCs) are environmental pollutants that mimic hormones and/or disrupt their function. Estrogenic EDCs (eEDCs) interfere with endogenous estrogen signalling pathway(s) and laboratory animal and human epidemiological studies have provided evidence for a causal link between exposure to them during embryonic/early life and neurological impairments. However, our understanding of the molecular and cellular mechanism(s) underlying eEDCs exposure effects on brain development, tissue architecture and function and behaviour are limited. Transgenic (TG) zebrafish models offer new approach methodologies (NAMs) to help identify the modes of action (MoAs) of EDCs and their associated impacts on tissue development and function. Estrogen biosensor TG zebrafish models have been applied to study eEDC interactions and resulting transcriptional activation (*via* a fluorescent reporter expression) across the entire body of the developing zebrafish embryo, including in real time. These estrogen biosensor TG zebrafish models are starting to deepen our understanding of the spatiotemporal actions of eEDCs and their resulting impacts on neurological development, brain function and behaviour. In this review, we first investigate the links between early life exposure to eEDCs and neurodevelopmental alterations in model organisms (rodents and zebrafish) and humans. We then present examples of the application of estrogen biosensor and other TG zebrafish models for elucidating the mechanism(s) underlying neurodevelopmental toxicities of eEDCs. In particular we illustrate the utility of combining estrogen biosensor zebrafish models with other TG zebrafish models for understanding the effects of eEDCs on the brain, spanning cellular processes, brain circuitry, neurophysiology and behaviour. Finally, we discuss the future prospects of TG zebrafish models as experimental models for studying more complex scenarios for exposure to contaminant mixtures on neurological development and function.

## 1 Introduction—Environmental Estrogenic EDCs

Estrogens are steroid hormones that play important roles in the development and physiological function of reproductive organs and a very wide range of other organs (Simerly et al., 1997; Wang et al., 2001; Wang et al., 2003; McCarthy, 2008; Bondesson et al., 2015). In vertebrates, the actions of estrogen are mediated by their binding to nuclear estrogen receptors (ERs: ERα and ERβ in rodents and human, ESR1, ESR2a and ESR2b in zebrafish) that regulates the transcription of estrogen-responsive genes (Bondesson et al., 2015). Estrogens can also bind to membrane-bound estrogen receptors, called G-protein coupled estrogen receptors (GPERs); activating the non-canonical estrogen signalling for intracellular calcium mobilisation, cAMP production and MAP kinase-mediated phosphorylation cascades (Périan and Vanacker, 2020).

**FIGURE 1:**
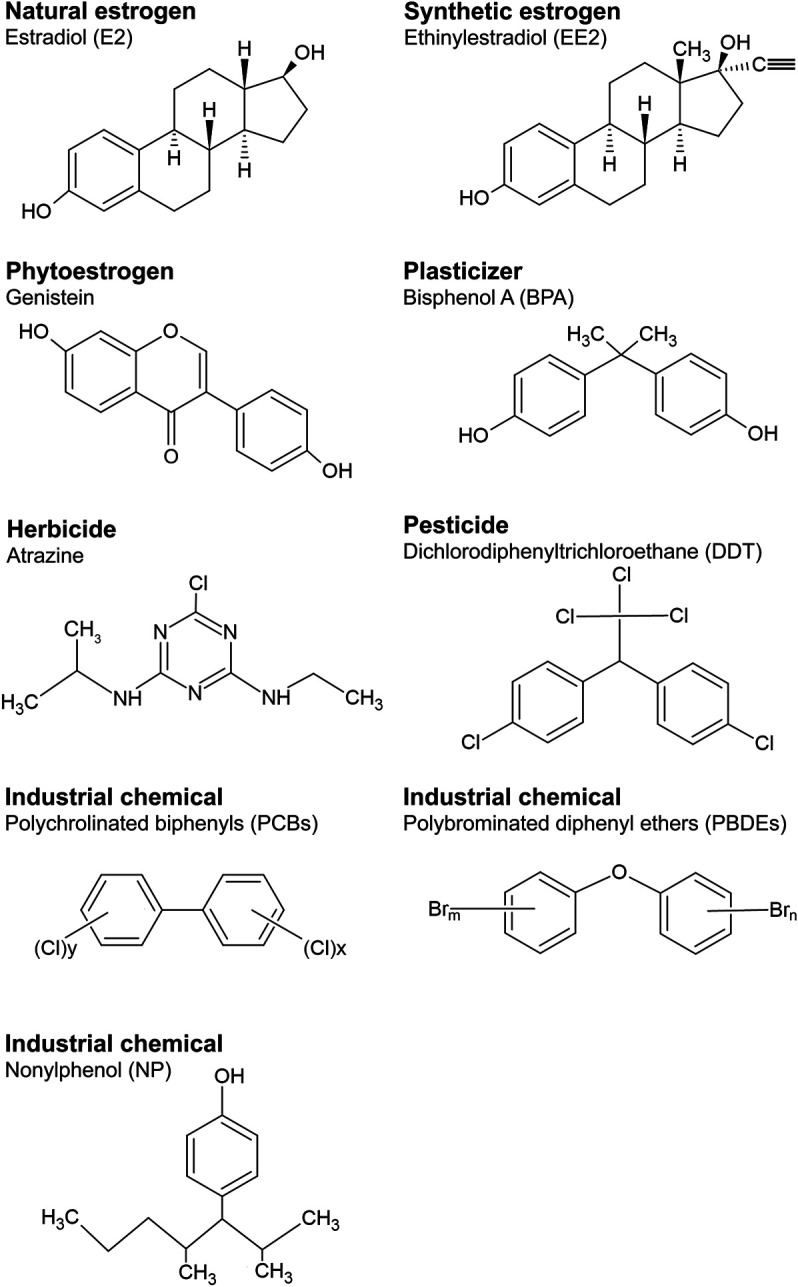
Structures of representative chemicals of natural and synthetic estrogens and eEDC subclasses.

A wide body of research has revealed that many exogenous substances can interfere with the actions of estrogens. These chemicals are referred as environmental estrogens or estrogenic endocrine disrupting chemicals” (eEDCs). In this review we adopt the definition of an “EDC” based on the World Health Organisation (WHO)/International Programme on Chemical Safety (IPCS) report in 2002, IPCS (2002) (International Programme on Chemical, 2002; Bergman et al., 2012), that states an EDC is “an exogenous substance or mixture that alters function(s) of the endocrine system and consequently causes adverse health effects in an intact organism or its progeny, or (sub)population.” Many substances used in industrial products are known to exert disrupting actions on endogenous estrogen pathways (Diamanti-Kandarakis et al., 2009; Gore et al., 2015; Tapiero et al., 2002). Figure 1 shows the chemical structures of some of these eEDC, that include synthetic estrogenic pharmaceuticals (e.g., ethinylestradiol (EE2; a component of contraceptive pills), plant estrogens (phytoestrogens, e.g., genistein), plasticizers, such as bisphenol A (BPA), herbicides (e.g., atrazine), various pesticides (notably dichloro-diphenyl-trichloroethane; DDT), and industrial chemicals including, polychlorinated biphenyls (PCBs), polybrominated diethyl ethers (PBDEs, flame retardants commonly used in furniture and housing materials) and surfactants such as nonylphenol (NP). Thus eEDCs are incredibly widespread in modern society. Indeed, more than 1,000 chemicals have now been identified with potential estrogenic activity based on collaborative estrogen receptor activity prediction project structure inventory (CERAPP) in the U.S. Environmental Protection Agency’s (EPA) ToxCast program (Richard et al., 2016). Many of these eEDCS are also highly resistant to biodegradation (Tapiero et al., 2002; Diamanti-Kandarakis et al., 2009; Adeel et al., 2017; Bergman et al., 2012) and their concentrations in the environment (i.e., soil, water and biota) are increasing (Adeel et al., 2017; Bergman et al., 2012).

The binding affinities of eEDCs (and their metabolites) to ERs have been shown to vary substantially (Cano-Nicolau et al., 2016; Kuiper et al., 1997; le Maire, 2010; Shanle and Xu, 2011). For some eEDCs, their binding affinities to ERs *in vitro*, however, do not always correspond with their relative estrogenic potency *in vivo* (Welshons et al., 2003). In some cases this is because eEDCs can affect estrogen functions through other ways, including *via* altering the levels of ER expression (Cao et al., 2012), ER turn-over (Masuyama and Hiramatsu, 2004), ER-mediated signal transduction (Routledge et al., 2000), endogenous estrogen synthesis (Jin et al., 2013) and/or clearance (Kester et al., 2000), as well *via* epigenetic mechanisms (Anway et al., 2005). Many eEDCs can also bind to GPER (Thomas and Dong, 2006; Périan and Vanacker, 2020) and to other nuclear receptors (NRs) including the androgen receptor (AR), estrogen related receptors (ERRs), thyroid hormone receptors (TRs), glucocorticoid receptor (GR), retinoid X receptors (RXRs), aryl hydrocarbon receptor (AhR) (Bondesson et al., 2015; le Maire, 2010; Shanle and Xu, 2011). Thus, MoAs for some “eEDCs” are diverse and can be far more complex than simply *via* directly affecting the nuclear ER-mediated signal transduction and estrogen hormone biosynthesis pathways (Bonefeld-Jørgensen et al., 2001; Périan and Vanacker, 2020).

Effects arising from exposure to eEDC in wildlife range from disruptions in gonad development, altered immune function and defects in reproduction and social and sexual behaviours. In some cases population level impacts (declines) have been implicated with these exposure effects (Bergman et al., 2012; Hotchkiss et al., 2008). In humans too, exposure to eEDCs have been linked with a range of reproductive abnormalities including alterations in sperm quality, fertility and puberty, and also with cancer, immune deficiency, obesity and both neurological development and neurological function (Bergman et al., 2012; Diamanti-Kandarakis et al., 2009; Gore et al., 2015). A recent environmental risk assessment highlighted that steroidal estrogens, EE2 and levonorgestrel (used in the emergency contraceptive pill), pose potentially the greatest environmental risk among nearly 1,000 approved drugs targeting human proteins (Gunnarsson et al., 2019). Furthermore, the impacts of some eEDCs have been shown to occur across multiple generations (Jang et al., 2012; Derouiche et al., 2015; Volkova et al., 2015; Brehm and Flaws, 2019).

There is a general consensus that embryonic/early life stages are the most susceptible to eEDC-related toxicities (Diamanti-Kandarakis et al., 2009; Gore and Crews, 2009; Gore et al., 2015). Given that estrogen signalling pathways are critically involved in the development of a wide variety of tissue and organ processes, including in the brain, in animals and humans (McCarthy, 2008; Bondesson et al., 2015), eEDC exposure during embryonic/early life may result in a wide range of developmental alterations, physiological dysfunctions and/or diseases and behavioural impairments in later life (Bergman et al., 2012; Diamanti-Kandarakis et al., 2009; Gore et al., 2015; Gore and Crews, 2009; Kuiper et al., 1997). Despite evidence for causal links between eEDC exposure and developmental neurotoxicity this is relatively poorly studied when compared with other effects of eEDCs (Legradi et al., 2018). In human too, there are relatively few epidemiological studies that have investigated links between early life exposure to an eEDC and neurodevelopmental phenotypes (Pinson et al., 2016; Nesan et al., 2018). There are substantial knowledge and methodological gaps in our understanding of the molecular mechanisms underlying developmental neurotoxicity of eEDCs and functional consequences in later life. The collective evidence for potential exposure effects of eEDCs on embryonic brain development in animals, including humans illustrates the urgent need for establishing effective and sensitive NAMs to more effectively evaluate their hazards and potential risks for neurological development and function.

In this review, we present the current knowledge on eEDC-related developmental neurotoxicity in model organisms (rodents and zebrafish) and humans, identifying the knowledge gaps and how zebrafish TG models can be applied to help fill them. We illustrate how applications of zebrafish TG models can help to verify the developmental neurotoxicity of eEDCs, through demonstrating links between eEDC exposure and the outcomes in brain anatomy, neurophysiology, brain function and behaviours. A major purpose of this review is to illustrate the utility of zebrafish TG models as a versatile and effective NAM for testing developmental neurotoxicity of eEDC and for providing new insights into the effects of eEDCs on brain development.

## 2 Effects of eEDCs on Embryonic Brain Development

### 2.1 Roles of Estrogen in Embryonic Brain Development—Evidence in Rodent Models

To understand the developmental neurotoxicity of eEDCs, we first detail the roles of endogenous estrogen in embryonic brain development. The underlying molecular mechanisms relating to estrogen fuctions in brain development have been most intensively studied in rodent models. Here it has been known for some time that the rodent embryonic brain is more strongly associated with estrogen activity than with that in the brain of later life stages. Reflecting this, the highest levels of estradiol (E2: the most potent endogenouse estrogen), aromatase (estrogen synthesising enzyme) activity and expression of ERs (ERα and ERβ) occur in the prenatal brain (George and Ojeda, 1982; McCarthy, 2008; Konkle and McCarthy, 2011) implying high importance of estrogen signalling in embryonic brain development. This is further supported by neurodevelopmental phenotypes in *ER*- and aromatase knock-out (KO) mice, where *ER*αKO mice fail to establish sexually dimorphic cell populations in the hypothalamic-pituitary-gonadal (HPG) axis (Simerly et al., 1997; Stephens et al., 2016). Developmental defects in HPG axis in *ER*αKO mice have also been shown to lead to alterations in sexual behaviour in later life, infertility in both males and females (Ogawa et al., 1997; Ogawa et al., 1998) and a drastic reduction in male-typical emotional/aggressive behaviours in males (Ogawa et al., 1997). These data show that ERα-mediated signalling pathway(s) play key roles in the development of sexually dimorphic circuitry and in the expression of reproductive and emotional behaviours. On the other hand, *ER*βKO mice show only modest reproductive phenotypes (Krege et al., 1998) but here marked defects occur in the neural cell migration of somatosensory cortex (Wang et al., 2001; Wang et al., 2003) and in development of calretinin-positive gamma-aminobutylic acid (GABA) ergic interneurons in the hippocampus, thalamus, and amygdala in the developing embryonic brain (Fan et al., 2006). Furthermore, embryonic stem cells from *ERβ*KO mice show defects in proliferation/self-renewal of neural stem cells as well as alterations in lineage specification of neural precursor cells into dopaminergic and serotonergic neurons *in vitro* (Varshney et al., 2017). These data strongly suggest that ERβ is essential for neural cell migration and differentiation/lineage specification. The estrogen synthesising enzyme, aromatase, has also been shown to play an organisational role in brain development (Bakker et al., 2002; Bakker et al., 2004). In both males and females, *aromatase* KO mice show several features of impairment in olfactory function and marked defects in reproductive behaviours. Notably, long term treatment with estrogen in adulthood (>6 weeks) was not found to restore the induced defects for investigating volatile odour in *aromatase* KO females. These animals also failed to display lordosis behaviour that can be induced by estrogen treatment in adult wild type females (Bakker et al., 2002). On the other hand, estrogen treatment in *aromatase* KO males has been shown not to restore the olfaction investigation behaviour but largely corrected deficits in coital behaviour (Bakker et al., 2004). This indicates that the reproductive behavioural phenotypes in *aromatase* KO males mostly result from a lack of estrogen-mediated hormonal input. Collectively, aromatase activity and estrogen are required for the development of the brain circuitry responsible for normal expression of olfaction-linked sexual behaviours in the female mouse which may be less prominent in the male (Bakker et al., 2002; Bakker et al., 2004).

Estrogen responsive element (ERE)-luciferase transgenic mice have further evidenced the importance of estrogen signalling in embryonic brain development. In this model, the ER-mediated transcriptional activation was observed predominantly in the ectodermal organs (i.e., the brain and the skin) from stage E12.5, while very little ER-activity was detected in other germ layer derived organs at the same prenatal stage (Della Torre et al., 2018). There was no apparent difference in such ectodermal ERE-luciferase activity in male or female at prenatal stage. These data again indicate that activation of estrogen signalling pathway occurs predominantly in the developing embryonic brain during the early embryonic stages. However, the detailed molecular mechanisms on which cell types the estrogen works, the estrogen receptor sub-types involved, and what the downstream target genes are that form part of the early neural ectodermal tissues in the estrogen biosensor mouse model have not been identified.

In addition to the canonical estrogen-mediated pathways described above, activation of the GPER, either by estrogen or by the specific agonist, G-1, has been shown to rapidly increase hippocampal CA1 spine density and memory consolidation in the adult mouse brain (Gabor et al., 2015), which are likely to be mediated by actin polymerisation through GPER-dependent JNK-cofillin phosphorylation (Kim et al., 2019). *GPER*-KO mice also showed some degree of sex-specific alterations in behavioural responses. For example, male *GPER*-KO mice showed less anxious behaviour in exploration tests while *GPER*-KO female mice showed altered stress-coping behaviours (Kastenberger and Schwarzer, 2014). Thus, in addition to the classical estrogen/ER-mediated transcriptional regulation, estrogen can also affect dendritic spine morphology and memory, anxiety and stress coping behaviours *via* a non-transcriptional mechanism. However, whether such GPER-mediated estrogen signalling pathways play a contributing role in embryonic brain development is currently unknown.

### 2.2 Roles of Estrogens in Embryonic Brain Development in Zebrafish

Estrogen signalling pathways in zebrafish are highly homologous to those established for rodents and humans. Zebrafish have three isoforms of ERs (as opposed to two in mammals), ESR1, ESR2a and ESR2b (previously denoted ERα, ERβ2 and ERβ1, respectively), and two isoforms of aromatase (Cyp19a1a and Cyp19a1b). The expression of ERs in the adult zebrafish is very much in common with that in rodents and humans, including in the liver, the gonad, the brain and the pituitary (Menuet et al., 2002). In addition, gene knockout of all three isoforms of *esrs* or gonad-specific aromatase (*cyp19a1a*) in zebrafish embryos collectively confirm their roles in differentiation and development of female reproductive organs (i.e., defects in follicle development in the ovary) (Lau et al., 2016; Lu et al., 2017), similar to that for *ERα*- and *aromatase* KO mice. Whether *esrs*- or *aromatase* KO in zebrafish manifests in any alterations in neurodevelopment, as occurs in rodent models, has not been established. Nevertheless, various lines of evidence strongly indicate estrogen signalling pathways are likely to play critical roles in embryonic brain development in zebrafish. Firstly, *esr* genes, particularly *esr2a* and *esr2b*, are expressed in diencephalon (for *esr2b*) and in telencephalon, preoptic area, hypothalamus (for *esr2a*) in developing zebrafish brain (Mouriec et al., 2009). Their expression levels in the embryonic brain start to increase from around 24 h post fertilisation (hpf) (at the onset of neurogenesis in the zebrafish embryo) to 48 hpf (when organogenesis completes and the embryo start to hatch) (Mouriec et al., 2009). This timing temporally correlates also with the induction of *cyp19a1b* gene, a brain specific aromatase gene (Mouriec et al., 2009). The spatiotemporal elevation in *esr2a* and *esr2b* (the homologs of ERβ) and *aromatase* expression levels during the course of the embryonic brain development are similar to that seen in the brain of mouse embryo (Harada and Yamada, 1992; Fan et al., 2006). It is important to note also that gonadal sex differentiation and sex dimorphism in the brain in zebrafish occur at much later life stages, at around 20–25 days post fertilisation (dpf) (Uchida et al., 2002; Lau et al., 2016) and between 20–40 dpf (Lee et al., 2017), respectively, compared with in mice. Thus, the roles of estrogen in embryonic and early larval stages in zebrafish are likely independent of its functions in sex differentiation.

GPER has also been suggested to play critical roles in zebrafish embryonic brain development. Whole mount *in situ* hybridization analyses revealed that *gper* mRNA is predominantly expressed across mid-brain regions in 36 hpf embryo and, by 72 hpf, is more intensified in the trigeminal ganglia (Shi et al., 2013). *gper* knockdown by morpholino during embryo development results in a deformed or small sized brain (at 24 hpf), and defects in axonal development and sensory neuron development (at 48 hpf stage) (Shi et al., 2013). Romano et al. have proposed that estrogens-GPER in the brain (i.e., pituitary) controls thyroid hormone signalling pathway to regulate heart rate in zebrafish embryo (Romano et al., 2017).

### 2.3 Developmental Neurotoxicity of eEDCs in Humans and Rodent Models

#### 2.3.1 Evidence in Humans

Epidemiological studies in humans collectively support a correlation between eEDC exposure *in utero,* or in early life more generally, and various kinds of behavioural problems in later life (Pinson et al., 2016; Walker and Gore, 2017; Nesan et al., 2018). This includes significant links between perinatal exposure to BPA and various types of neurobehavioural phenotypes in childhood, notably hyperactivity, aggressive behaviour, anxiety, depression, lack of attention, and memory and/or cognitive impairments (Casas et al., 2015; Pinson et al., 2016; Nesan et al., 2018). Prenatal exposures to some persistent eEDCs (e.g., PCBs and PBDEs) have also been shown to correlate with difficulties in attention and executive functions (Verner et al., 2010; Pinson et al., 2016; Nesan et al., 2018). Importantly, in most of these epidemiological studies, correlations observed between eEDC exposure and behavioural phenotypes have been particularly evident for children exposed *in utero*. Hazardous influences of eEDC exposure on brain development have therefore been implicated in the increased prevalence of some of neurodevelopmental diseases in human, including attention-deficit hyperactivity disorder (ADHD) and autism spectrum disorders (ASDs) that are known to exhibit a marked gender bias (Pinson et al., 2016; Nesan et al., 2018). In a systematic literature search of the published clinical and epidemiological studies, three eEDCs, namely PCBs, DDTs, and PBDEs, have been classified as “developmental neurotoxicants” for humans, together with eight other substances (e.g., lead, methylmercury, toluene) (Grandjean and Landrigan, 2014). However, given these pollutants can affect multiple NR signalling pathways, further research would be required to validate the link between their neurotoxicity and a responsible signalling pathway.

#### 2.3.2 eEDC-Induced Developmental Neurotoxicity in Rodent Models

In agreement with human epidemiological data, emerging evidence from rodent models have also shown that pre- and post-natal exposure to eEDCs can induce a range of behavioural defects, including alterations in cognition, memory, emotional control and reproductive behaviour. Such behavioural phenotypes are highly related to changes in brain anatomy, neuronal cell population/morphology, neuronal circuitry and gene/protein expression profiles in specific brain regions during brain development, most notably in the sexually dimorphic HPG axis and in the hippocampus (Parent et al., 2011; Lopez-Rodriguez et al., 2021).

- Effects of eEDCs on HPG axis development

Aligning with neurodevelopmental phenotypes in *ERα*- and *aromatase* KO mice, estrogen signalling pathways are known to be critically involved in development of HPG axis, wherein defects during development result in alterations in puberty, emotional responses and reproductive behaviour. ERs are expressed in various cell types in the HPG axis and estrogen transduces multiple signals to control neural excitability, peptide release and gene expression (Acevedo-Rodriguez et al., 2018). As such the molecular mechanisms involved in HPG axis development are likely to be one of the most vulnerable targets for eEDC neurotoxicity. Oral administration EE2 during the perinatal period (from embryonic day 10 to postnatal day 40) has been shown to induce alterations in reproductive development, including advanced vaginal opening and a shorter estrus cycles (Derouiche et al., 2015). Concurrently, these developmental EE2 exposures resulted in increased numbers of gonadotropin-releasing hormone (GnRH) neurons (for pharmacological doses) and altered the distribution of GnRH neurons (for environmental doses). In addition, developmental exposure to EE2 has been shown to impair female-typical reproductive behaviours (e.g., a disturbed maternal behaviour, a higher lordosis response) and also increased anxiety-related behaviours (Derouiche et al., 2015). Fetal exposure to low doses of BPA has been shown to induce aggressive behaviour and reduce the relative testis weight (per Gram of body weight) in 8 week old F1 males (Kawai et al., 2003). These data support the hypothesis that developmental processes associated with neuroendocrine circuits in the HPG axis are vulnerable to eEDC exposure effects that can lead to changes in development of reproductive organs, reproductive and/or sex-specific emotion-related behaviours.

The administration of Aroclor 1221 (a commercial PCB mixture), at a dosing relevant to environmental exposures, to pregnant rats during gestation has been shown to reduce the expression levels of ERβ and ERα, and reduce both kisspeptin fibre density and GnRH neuron activation (detected by co-staining of c-Fos and GnRH neuron antibodies) in the Antero Ventral Peri-Ventricular nucleus (AVPV)—a sexually dimorphic small cell cluster in the preoptic area of the hypothalamus—in the female offspring (Salama et al., 2003; Dickerson et al., 2011). Similarly, the administration of genistein to new born rats for the first 4–5 days, at a dose equivalent to the total amount of isofravones ingested by infants fed soy formula, has been shown to reduce the number of neuroactive GnRH neurons and kisspeptin fibre density in the AVPV in females (Bateman and Patisaul, 2008). These female-specific alterations in HPG axis development were accompanied by advanced vaginal opening, dysregulation in estrous cycle and impairment in reproductive behaviour (Bateman and Patisaul, 2008). Collectively, these data strongly suggest that early life exposure to eEDCs can be particularly disruptive to HPG axis development thereby affecting reproductive development and behaviour.

- Effects of eEDCs on hippocampus development

There are various data demonstrating eEDC exposure *in utero* or in early-life results in impairments in hippocampal development and leads to deficits in cognitive function, memory and mood control in later life. For instance, female mice (F0) exposed *via* the diet to BPA from preconception until lactation at a dose representative of human exposure levels have been shown to induce the male-specific depression-like behaviour (time spent immobile) (Xin et al., 2018). Such behavioural alterations following developmental BPA exposure correlated with disrupted hippocampal neurotransmitter systems, in particular, a reduction in hippocampal serotonin in the F1 offspring (Xin et al., 2018). Oral administration of Arochlor 1254 (a PCB mixture) to dams during the perinatal period (from the sixth day of gestation to postnatal day 21, p21) has been shown to cause a modest reduction in the number of new born neurons in hippocampus in adult stage (p56) and to inhibit the developmental increase (between p21 and p28) in spontaneous excitatory postsynaptic currents (sEPSCs) frequency in the dentate gyrus slice of F1 mice (Parent et al., 2016). These data suggest that A1254 may be particularly harmful to the maturation process of excitatory synapses in new born granule cells in the hippocampus. It has been suggested that the disruption of thyroid receptor signalling may be involved in the neurotoxic effects of A1254 for perinatal exposures as evidenced by hypothyroxinemia (reductions in thyroid hormone T4 levels) at p21 (Crofton et al., 2000). However, other mechanisms have also been suggested to contribute to this phenotype given that T4 level was normal by p56 following perinatal exposure to A1254 (Parent et al., 2016). These rodent data may link to cognitive defects observed in children that have been exposed to PCBs in their perinatal period (Verner et al., 2010).

### 2.4 eEDC-Induced Developmental Neurotoxicity in Zebrafish Models

The most well-studied effects of eEDCs on fish are their impacts on development and function of reproductive organs, where they can lead to sex reversal, defects in reproductive tissues/organs, alterations in reproductive behaviour and full or partial infertility, as illustrated in many different fish species, including zebrafish (Mills and Chichester, 2005). However, eEDCs are also known to induce various alterations in brain development and behaviour in fish. For instance, developmental exposures to low-dose BPA (0.0068 μM, 1,000-fold lower than accepted human daily exposure) and bisphenol S (BPS), a common analogue used in “BPA-free products,” during the critical window of hypothalamic neurogenesis [e.g., from 24 hpf to 48 hpf] have been shown to induce hyperactive behaviour phenotypes in five dpf zebrafish larvae (Kinch et al., 2015). This behaviour phenotype appeared to relate to precocious neurogenesis in the hypothalamus, whereby BPA/BPS exposure resulted in an increase in the number of neurons in the hypothalamus. Interestingly, this early neurogenesis phenotype in BPA/BPS exposed embryos was blocked by the aromatase inhibitor, fadrozole, by aromatase knockdown (aromatase morpholino) and by an AR antagonist (flutamide), but not by ER antagonist/ICI182,780. These data suggest that the neurodevelopmental phenotypes induced by BPA/BPS exposure may be mediated *via* aromatase and AR-dependent non-canonical pathway rather than by the canonical ER-signalling pathway (Kinch et al., 2015).

Developmental exposure of zebrafish embryos [e.g., from 1 dpf to five dpf] to low doses of EE2 (e.g., 0.5 nM) and NP (a surfactant known to be weakly estrogenic, e.g., 0.5 μM) resulted in an increase in GnRH neuron number and GnRH neuron fibres in specific brain regions, including the olfactory bulb, telencephalon, anterior commissure, preoptic area and hypothalamus, in five dpf zebrafish embryo/larvae (Vosges et al., 2010; Vosges et al., 2012). Under the same exposure conditions, brain specific aromatase in zebrafish, *cyp19a1b*, was strongly induced in radial glia cells, that localise with GnRH neurons (Vosges et al., 2010; Vosges et al., 2012). However, how/whether *cyp19a1b* induction in adjacent radial glia cells affects the number of GnRH neurons in hypothalamus or whether this change in GnRH neuron ontogeny affects reproduction and/or behaviour in later life has not been established.

Exposure of zebrafish to low-doses of EE2, from 0 to 80 dpf, have been shown to induce anxiety behaviour and increased shoaling intensity (indicating stress) (Porseryd et al., 2017) as well as defects in fertilisation success in F0 fish of both sexes (Volkova et al., 2015). Such behavioural phenotypes were shown to persist even after a long (120 days) depuration period in clean water and was furthermore seen in the F1 offspring of both sexes (Volkova et al., 2015; Porseryd et al., 2017). This suggests that developmental exposure to EE2 caused permanent alterations in brain circuitry and function. Adding to this, brain transcriptome analyses revealed differential expression of genes involved in synaptic vesicle development/function, circadian rhythm, cytoskeleton, cell adhesion in female and those related to cholesterol synthesis and synaptic proteins in male in EE2 exposed animals (Porseryd et al., 2017). Notably, *synaptic vesicle glycoprotein 2b* (*sv2b*), was the only gene whose expression was affected by the developmental exposure to EE2 in both sexes. Together, these data suggest that eEDC exposure during a critical window of brain development may impact on neurogenesis and synaptogenesis, with consequences for permanent, and even transgenerational, influences on behaviour and fitness in fish.

### 2.5 Commonalities in eEDC-Related Developmental Neurotoxicity Across Animal Models and Knowledge Gaps

Developmental neurotoxicity for exposure to eEDCs is known for a wide variety of vertebrates. The commonalities seen in different species include: 1) the embryonic/early life stage brain is especially vulnerable to eEDC exposure as compared with the adult brain, 2) the behaviour phenotypes associated with eEDC exposure are often irreversible, suggesting developmental eEDC exposure can cause organisational defects in brain circuit/tissue development 3) eEDC-related developmental neurotoxicity can be observed across generations in model organisms, and 4) behavioural outcomes related to eEDC developmental neurotoxicity often do not become obvious until in later life. Such latency of behavioural outcomes, until recently, has limited our awareness of their potential hazards, but they are now associated with the increase in prevalence of neurodevelopmental abnormalities in animals, including humans. All of the above serves to highlight the urgent need for a more global approach to the assessment of eEDC-related developmental neurotoxicity (Hecker and Hollert, 2011; Legradi et al., 2018).

Major knowledge gaps hindering evaluations on the risk of an eEDC for neurodevelopment include the very limited information on their mechanisms of actions. As highlighted above eEDC can act at multiple sites and through various MoAs, and in brain region and developmental stage specific manners. This complexity has made it difficult to establish sensitive biomarkers that specifically signal for their developmental neurotoxicity. There are also still insufficient data demonstrating eEDC-related real-time changes in brain architecture, circuitry and neuronal excitability and behaviour across all life stages in animals including in humans. Such data is extremely difficult to acquire from commonly used existing assay systems for toxicology assessments, including cell based systems or rodent models. These shortfalls in information make it difficult to reliably link physiological and/or associated behavioural endpoints with developmental neurotoxicity of eEDCs. To date, especially in rodents, eEDC-induced developmental neurotoxicity has almost exclusively been focused on their effects on the hippocampus and sexual dimorphism in HPG axis and on behavioural changes associated with the developmental impacts on those brain regions (i.e., aggression, memory, cognition and reproductive behaviour). This, at least in part, is because the development of these brain tissues is known to be highly regulated by estrogen signalling (and other nuclear receptor) pathways. Until recently, the effects of eEDCs on development of other brain regions have received very little attention and as such there is a high likelihood that other important eEDC-related effects on brain development may have been overlooked. Mechanism-based *in vivo* assay systems that enable observation of real time effects on the entire process of embryonic brain development are much needed and TG zebrafish have great potential for this as we now illustrate.

## 3 TG Zebrafish as NAM for Assessing eEDC-Related Developmental Neurotoxicity

### 3.1. Zebrafish Models in Neurodevelopmental Research

The zebrafish has become an important vertebrate experimental model for studies on neurodevelopment, and for a number of good reasons. Firstly, the embryos they produce are transparent and develop rapidly allowing for the application of methods for visualising processes of embryonic brain development (including the whole brain) in real-time. This is not feasible in almost all other vertebrate models (with the exception of amphibians). Secondly, the brain of zebrafish embryo shows considerable similarity in the ontogenetic path, anatomy, physiology and genetics to that in mammals, including humans. All the fundamental cell populations, neurotransmitters and their receptors, neuroendocrine hormones, enzymes involved in synthesis/metabolism of neuropeptides/hormones, and a large part of brain morphology in mammals are well conserved in the zebrafish. Indeed there is very extensive research that strongly supports the translational relevance of zebrafish models in neurotransmitter-mediated brain functions, neuroendocrine responses and various types of associated behaviours: including anxiety, depression, memory, cognition and social behaviour (Kalueff et al., 2014; Nishimura et al., 2015; Legradi et al., 2018; Sakai et al., 2018). Thirdly, genetic manipulation of the zebrafish embryo is relatively easy and many useful neuronal cell type-specific TG lines, biosensor lines, gene KO and mutant lines are now available within the research community. Lastly, the fundamental stages of development of the central nervous system (CNS) are completed within 5 days of fertilisation in the zebrafish, before it is categorised as a “regulated animal” under UK home office guidance for animal testing and research (Office, 2021) and EU Directive 2010/63/EU on the protection of animals used for scientific purposes (Strähle et al., 2012). Thus, neurodevelopmental research using zebrafish embryo aligns also with the demand for Replacement, Reduction and Refinement of the use of animals in research (the 3Rs).

Although the zebrafish embryo provides a highly versatile *in vivo* model for research on genetic neurodevelopmental disorders and chemical induced-developmental neurotoxicity (Kalueff et al., 2014; Nishimura et al., 2015; Legradi et al., 2018; Sakai et al., 2018), it should be recognised also that there are clear anatomical differences between the brain of mammals and the zebrafish. This is especially the case in the forebrain which is the centre of most cognitive functions. In the zebrafish, the forebrain is comprised of the telencephalon (cortex and sub-cortex regions in the mammals, pallium and sub-pallium in the zebrafish) and diencephalon (the pineal, the habenula and the thalamus). The functional homolog of the mammalian hippocampus appears to be the antero-dorsolateral pallium in the zebrafish and the amygdala nuclei (e.g., central amygdala, basolateral/lateral amygdala) are not clustered but localised in the pallium and subpallium in the zebrafish (Cheng et al., 2014). Such anatomical differences need due consideration in neurodevelopment studies, especially regarding functional circuit analyses when using the zebrafish brain.

### 3.2 Estrogen Biosensor TG Zebrafish as Models for Assessing the Impacts of eEDC Exposure on Genomic Estrogen Functions

#### 3.2.1 Estrogen Biosensor TG Zebrafish Models

Several estrogen biosensor TG zebrafish models have been established that enable studies into real-time cell and tissue responses to natural estrogens and eEDCs. Most of them have been generated by random genome insertion of an estrogen responsive reporter transgene. More recently, targeted knock-in of the reporter genes into the promoter region of *vitellogenin 1* (*vtg1*: a well-known biomarker for eEDC exposure) by CRISPR/Cas9 technology was employed to generate *vtg1:green fluorescent protein (GFP)* line (Abdelmoneim et al., 2020). In all cases, the expression of the reporter transgene (i.e. fluorescent proteins or luciferase) is under control of either 1) the endogenous promoter/enhancer of an estrogen inducible gene: e.g. *vitellogenin1* (*vtg1*) (Chen et al., 2010; Abdelmoneim et al., 2020), *cyp19a1b* (Tong et al., 2009), or 2) the synthetic tandem repeats of estrogen responsive element (ERE) which can be activated by the binding of estrogens/eEDCs and ERs (Legler et al., 2002; Gorelick and Halpern, 2011; Lee et al., 2012a; Lee et al., 2012b; Green et al., 2016; Moreman et al., 2017; Green et al., 2018; Moreman et al., 2018). These estrogen biosensors induce a reporter gene expression in response to steroidal estrogens and also eEDCs, which can be detected non-invasively *via* imaging. Thus, they provide spatial and temporal information of estrogen activity in live zebrafish (embryo/larvae and/or adults). The sensitivity and tissue responsiveness to estrogens and/or eEDCs in the different estrogen biosensor line varies depending on the promoters/enhancers used and the presence or absence of Gal4-UAS reporter amplification process. Tissue specific responsiveness observed in each different estrogen biosensor depends on the tissue specificity of the promoter/enhancer genes used. Ilustrating this, tg (*vtg1:GFP*) shows a specific tissue response to estrogens/eEDCs only in the liver (Chen et al., 2010; Abdelmoneim et al., 2020) whereas in tg (*cyp19a1b:GFP*) GFP induction by estrogens/eEDCs exposure occurs exclusively in radial glia cells in the zebrafish brain (Tong et al., 2009; Brion et al., 2012). On the other hand, estrogen biosensors carrying the synthetic ERE-dependent reporter (ERE:TG models) show estrogen responses across a wider range of organs, including the liver, heart, brain, pancreas, muscle somites and gonads (Gorelick and Halpern, 2011; Lee et al., 2012a). This may be because these ERE-TG models carry a transgene that consists of the tandem repeats of ERE in conjunction with a minimum promoter and fluorescent reporter sequences only. Thus they can detect ER-mediated transcriptional activities widely throughout the embryo without being affected by tissue specific enhancers or suppressor elements in the endogenous estrogen responsive genes.

It is important to note that in the embryonic/early larval stage, endogenous levels of estrogen activity are not optically detectable in any of estrogen biosensor lines that have been established to date. This may mean that estrogens are either not present or they are at levels in embryo/larval stages below the detection sensitivity limits of the estrogen biosensor lines and/or the used imaging systems. If any estrogen signalling is in operation in non-chemically exposed embryos, they are not responsive to the estrogen receptor antagonist, ICI182,780, indicating that any such signals are not mediated by estrogen/ER activation (Lee et al., 2012a; Brion et al., 2012; Takesono et al., 2022). Studies with various estrogen biosensor zebrafish lines however have demonstrated that estrogens and/or eEDC-induced estrogen responses occur in the developing embryonic brain as we now illustrate (also listed in Table 1).

**TABLE 1:** TG zebrafish models available for examining MoAs of eEDC-induced developmental neurotoxicity: Top groups, estrogen biosensors; second group, other biosensors; third group, TG models for functional brain imaging; fourth group, TG/gene-knockout/mutant models for testing integrated effects of eEDCs on developmental neurotoxicity: oxidative stress biosensor and examples of neurodevelopmental disease models or *robo2* ^*−/−*^ mutant.

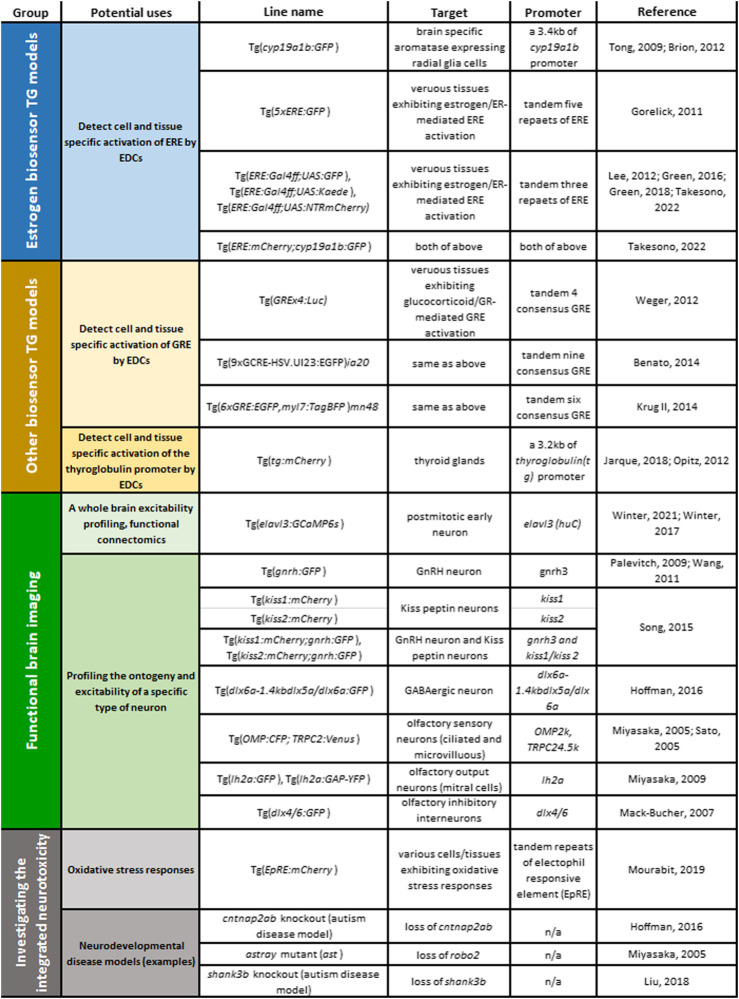

#### 3.2.2 Tg(cyp19a1b:GFP)

*Aromatase*, *cyp19* genes (*cyp19a1a and cyp19a1b*) in zebrafish are sensitive biomarkers for assessing the estrogenic potencies of natural estrogens and/or eEDCs (Jarque et al., 2019). In tg (*cyp19a1b:GFP*) zebrafish (*cyp19a1b* is a brain specific *aromatase* gene in zebrafish) GFP expression is under control of 3.4 kb of endogenous *cyp19a1b* promoter which includes an ERE and possibly also a glia specific enhancer sequence (Le Page et al., 2008; Tong et al., 2009). Thus, in tg (*cyp19a1b:GFP*) zebrafish, GFP expression is induced when ERE is activated *via* estrogen/ER-binding and it occurs only in radial glia cells (Brion et al., 2012; Tong et al., 2009). In five dpf tg (*cyp19a1b:GFP*) embryo-larvae, GFP expression occurs in radial glia cells of several brain regions, including the olfactory bulb, the telencephalon (pallium), the preoptic area and the hypothalamus, and is responsive to a wide variety of eEDCs including EE2, NP, BPA and its analogues, some pesticides and their metabolites [e.g., DDT and 2,2-bis(p-hydroxyphenyl)-1,1,1-trichloroethane, HPTE (a metabolite of methoxychlor)] and phyto-estrogens (e.g., genistein) (Brion et al., 2012; Cano-Nicolau et al., 2016). GFP-expressing radial glia cells locate along the midline ventricles and extend their characteristic long radial processes toward the pia matter, the most outer surface of the brain. For synthetic estrogens which are the most potent estrogenic compounds (e.q., EE2 and DES), the estrogen responses can be observed at environmentally relevant concentrations (e.q., EC50 of EE2, 0.013 nM). Tg (*cyp19a1b:GFP*) embryo also show induction of GFP in the same brain cells/regions in response to some androgens (e.g., 17α-methyl testosterone (MT)) and progesterons (e.g., norethindrone) but with much-lower sensitivity than natural estrogens, and this can be greatly inhibited by an ER antagonist/ICI182,780 but not by AR antagonist/flutamide (Brion et al., 2012). Thus, these responses to androgens and progesterons are likely mediated by ER-signalling pathway. The tg (*cyp19a1b:GFP*) embryo model has now been developed for use in a high throughput assay format (EASZY assay) to screen for estrogenic activity of pollutants in surface and wastewater samples and has furthermore very recently been accepted as an Organisation for Economic Co-operation and Development (OECD) Guideline test protocol for environmental estrogens (OECD test No. 250) (OECD, 2021).

The tg (*cyp19a1b:GFP*) has also been used to illustrate the spatiotemporal development of *cyp19a1b*-expressing radial glia over the course of zebrafish embryonic brain development. In zebrafish, GnRH3 neurons originate from the dorso-anterial region of the forebrain (near olfactory epithelia) at around 26 hpf and they migrate ventrally through the olfactory bulb, ventral telencephalon (subpallium) and preoptic area to reach the hypothalamus where an extensive network of GnRH neuron fibres extend widely across the CNS by 4-5 dpf (Abraham et al., 2008). This GnRH neuron development is critical for the establishment of HPG axis and is known to be regulated by ER-mediated pathway in both rodents and zebrafish. Interestingly, the ontogeny of GnRH3 neurons overlaps closely with that of *cyp19a1b*-expressing radial glia cells and they are consistently co-localised with each other (Vosges et al., 2010; Vosges et al., 2012). Given that radial glia cells are embryonic neural stem cells and function as scaffolds to guide the radial migration of new born neurons, it has been suggested that *cyp19a1b*-expressing radial glia cells carry out this role in GnRH3 neuron development (Vosges et al., 2010; Vosges et al., 2012). Notably, exposure to low doses of EE2 or NP has been shown to increase the number of GnRH neurons and GnRH neuron fibres and a concomitant induction of the estrogen synthesising *cyp19a1b* gene in the radial glia in five dpf tg (*cyp19a1b:GFP*) embryos (Vosges et al., 2010; Vosges et al., 2012). Thus, eEDCs may affect the function of radial glia by modulating *cyp19a1b* gene expression, that in turn impacts on normal neurogenesis and cell migration of GnRH neurons during embryonic brain development. Many studies using rodent models have demonstrated the importance of the interplay between glia and GnRH neurons in HPG neuroendocrine system controlling reproduction (Smedlund and Hill, 2020). However, whether the functional relationship seen between aromatase-expressing radial glia and GnRH neurons that occur in the embryonic brain development of fish are conserved in rodents and humans is currently not known.

#### 3.2.3 ERE:TG Lines

Two different ERE:GFP zebrafish, tg (*5xERE:GFP*) (Gorelick and Halpern, 2011) and tg (*ERE:Gal4ff*;*UAS:GFP*) (Lee et al., 2012a), have been established where the expression of the reporter transgene GFP is specifically driven by the activation of synthetic EREs through the binding of estrogens and/or eEDCs to ERs. Using these systems, a spatiotemporal profile of ER/ERE activation can be visualised by non-invasive microscopic approaches. The use of the tandem repeated short EREs in these models combined with a minimal promotor in the reporter genes allows for the detection of ER-mediated transcriptional activities throughout the embryo without being affected by tissue specific enhancers or suppressors. Among the estrogen responsive organs, the liver and the heart (i.e. the heart valves) have been shown to be especially sensitive to estrogens in both of ERE:GFP lines detecting responses for environmentally relevant exposures (Gorelick and Halpern, 2011; Lee et al., 2012a) similar to the minimum detection level seen for the radial glia in tg (*cyp19a1b:GFP*) embryo (Brion et al., 2012). Accordingly, these models have been used to assess contamination of surface- and waste-waters with environmental estrogens in the USA (Gorelick et al., 2014), across Europe (Brion et al., 2019) and England (Cooper et al., 2021a; Cooper et al., 2021b).

For exposures to higher concentration of estrogen (e.q., 100 μg/L E2), tg (*5xERE:GFP*) embryos also showed several other uncharacterised estrogen responsive cell populations in the brain: in the preoptic area, the hypothalamus and the olfactory area. Using tg (*ERE:Gal4ff*;*UAS:GFP*) embryos exposed to 25–100 ng/L EE2, we have also found another estrogen responsive cells in the olfactory bulbs, the most anterior brain tissues involved in the olfaction (Takesono et al., 2022). Importantly, an ER antagonist/ICI182,780 treatment markedly inhibited GFP expression in all of these estrogen responsive cells in both ERE:GFP models, indicating these responses are indeed mediated by ER/ERE activation.

In our laboratory we have established several further different ERE:TG zebrafish lines for investigating responses to eEDCs. They include a line generated through crossing tg (*ERE:Gal4ff*;*UAS:GFP*) with a pigment free mutant zebrafish Casper (Green et al., 2016), a line where the *UAS:GFP* has been substituted with another *UAS: reporter* gene*, Kaede* (Green et al., 2018), a line tg [*ERE:Gal4ff*;*UAS:nitroreductase* (NTR)-*mCherry*] (shortened name *ERE:mCherry*), that enables NTR-mediated chemical/genetic cell ablation, and a double transgenic line carrying both *ERE: mCherry* and *cyp19a1b:GFP* reporter genes, tg (*ERE:mCherry*;*cyp19a1b:GFP*) (Takesono et al., 2022) (listed in Table 1). The tg (*ERE:Gal4ff*;*UAS:GFP*) in a pigment mutant Casper background substantially improves the visual clarity in estrogen responsive GFP expression *in vivo* as compared with the tg (*ERE:Gal4ff*;*UAS:GFP*) in wild type background and has facilitated the detection sensitivity for the study of target tissues and potencies of a wide range of estrogenic chemicals (Green et al., 2016; Green et al., 2018) and their EDC mixtures (in surface wastewaters) (Cooper et al., 2021a; Cooper et al., 2021b). In the tg (*ERE:Gal4ff*;*UAS:Kaede*) line the reporter protein, Kaede, can be photo-converted from green fluorescent to red fluorescent with a short UV exposure and this allows for time-sequenced and tissue specific ER/ERE-responses to be monitored at a desired timing or location in the live embryo-larvae. This system is particularly useful for assessing interactions between different estrogenic chemicals (Green et al., 2018) and may also be applied for cell lineage tracing of estrogen responsive cells during the course of organ growth and development. In the model tg (*ERE:mCherry*), NTR-mediated chemical/genetic cell ablation is under control of estrogen-mediated ERE-activation that enables selective ablation of estrogen responsive cells through exposure to estrogen (to drive ERE:Gal4ff activation and subsequently to induce NTR-mCherry expression) together with a prodrug metronidazole (MTZ) to induce a cytotoxic metabolites only in estrogen responsive cells. Through selecting the timing of estrogen and MTZ exposure, cell ablation of estrogen responsive cells at a specific ontogeny can be achieved (Takesono et al., 2022). These ERE:TG models provide systems for further characterising various types of cells specifically responding to exogenous exposure of estrogens/eEDCs and to examine whether estrogen activity in these cells is important in the tissue/organ development/function, and if so, what implications this may have for exposure to eEDC.

**FIGURE 2:**
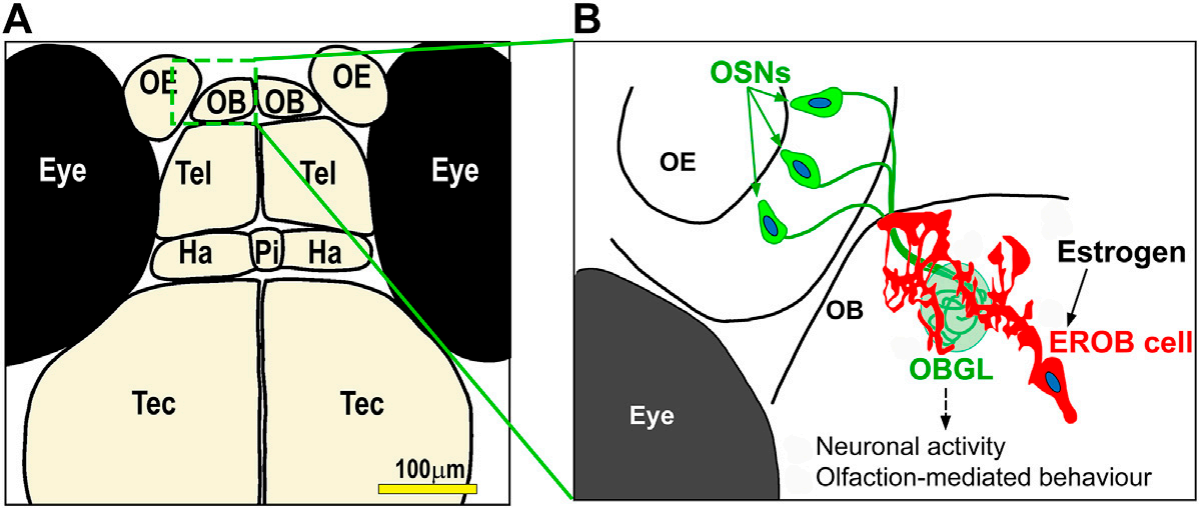
A model for estrogen-mediated regulation of development of olfactory sensory system in zebrafish embryo. **(A)** Illustration of the forebrain regions of four dpf zebrafish embryo/larvae. Olfactory epithelia, OE; olfactory bulb, OB; Tel, telencephalon; Ha, habenula; Pi, pineal; Tec, tectum. **(B)** A cartoon for estrogen responsive olfactory bulb (EROB) cell in four dpf zebrafish embryo/larvae. From the early onset of embryo development (i.e., 27 hpf), estrogen acts on a specific glia in the OB (estrogen responsive olfactory bulb (EROB) cells), inducing estrogen/ER-mediated transcription. The EROB cell in turn regulates olfactory glomerular development through tight interaction with projections of olfactory sensory neurons (OSNs) at the OB glomeruli (OBGL). We have shown that this exposure to estrogen during early life affects the EROB cells altering the local excitability of the OB, eventually leading to a defective olfaction-sensing capability in later life (Takesono et al., 2022).

Most of estrogen responsive cells which have newly been identified using estrogen biosensor lines remain largely uncharacterised. An exception to this is for an estrogen responsive cell type we have recently discovered in the brain. Using tg (*ERE:Gal4ff*;*UAS:GFP*) and tg (*ERE:mCherry*;*cyp19a1b:GFP*) zebrafish embryos, we have shown that the olfactory bulb (OB) is one of the earliest target tissue for estrogen in the zebrafish embryo, with estrogen-mediated transcriptional activation occurring specifically in a small number of cells in this brain tissue from early neurogenesis stage (i.e., 27 hpf) (Takesono et al., 2022). The OB is located in the anterior-most part of the forebrain and is the primary centre for processing olfactory signals. By immunohistochemical analyses, we found that these estrogen responsive cells in the OB are a type of glia, which we name estrogen-responsive olfactory bulb (EROB) cells (Figure 2). The ontogeny of EROB cells coincides with a critical point in the timing of olfactory glomerular (the first synaptic neuropils in the OB) development. In response to estrogen, EROB cells extend ramified projections that overlay the outermost layer of the OB and interact closely with olfactory sensory neurons (OSNs) at the olfactory glomeruli. Inhibiting estrogen activity by exposure to an ER antagonist, ICI182,780 (ICI), and/or EROB cell ablation using NTR/MTZ-mediated cell ablation system impedes olfactory glomerular development, including the topological organisation of olfactory glomeruli and inhibitory synaptogenesis in the OB. These data indicate that estrogen and EROB cells are critically involved in olfactory glomerular development. Activation of estrogen signalling specifically inhibits both intrinsic and olfaction-dependent neuronal activity in the OB, while ICI or EROB cell ablation results in the inverse effect on neuronal excitability to that of estrogens. Furthermore, altering estrogen signalling disrupts olfaction-mediated behaviour in later larval stages. These data suggest that estrogens act on glia to regulate development of OB circuits, thereby modulating the local excitability in the OB and olfaction-mediated behaviour (Takesono et al., 2022). This also highlights the possibility for this estrogen/EROB cell cascade in early embryonic stages being potentially an important site of action for eEDCs. To our knowledge, such implications of eEDC-related developmental neurotoxicity in cellular processes regulating development of olfactory sensory system have not been reported previously. Given the high conservancy in estrogen signalling pathways and also conservancy in the molecular/cellular processes regulating olfactory development in animals and human, olfactory sensory development may be an important target of eEDC-related developmental neurotoxicity in vertebrates more generally. In fact, similar instructive roles of embryonic glia in the foundation of OB glomeruli have previously been suggested by descriptive histological analyses using rat embryonic brain sections (Valverde et al., 1992; Ramon-Cueto and Valverde, 1995; Bailey et al., 1999). It would be important to test whether such embryonic glia in the OB in rodent models can similarly respond to estrogens as EROB cells do in the zebrafish embryo.

### 3.3 A Note of Caution on “eEDC” Effects and Other Biosensor Models Available for eEDC Target Pathways

It should be emphasised that some chemicals classified as eEDCs can bind to and activate various other types of NRs, including AR, ERRs, TRs, GR, RXRs, and AhR and they can also affect cross-talk of these NR-mediated signalling pathways. Examples of such eEDCs include various phthalates, alkylphenols (e.q., NP), tributyltin (TBT), DDT, BPA, PCBs and more (Bondesson et al., 2015; le Maire, 2010; Shanle and Xu, 2011). In developing a clear picture of how “eEDCs” affect embryonic brain development it is important to distinguish between those effects mediated directly *via* ERs *versus* these other NR-mediated responses. To date, several different biosensor TG zebrafish lines have been developed to study GR- (Benato et al., 2014; Krug II et al., 2014; Weger et al., 2012) and TR- (Jarque et al., 2018; Opitz et al., 2012) signalling pathways and used to assess chemical impacts of EDCs on these signalling pathways (also listed in Table 1). For example, using the tg [*Glucocorticoid Response Element* (*GRE*)*x4:Luc*] embryo (named Glucocorticoid Responsive *In vivo* Zebrafish Luciferase activitY assay, GRIZLY assay) TBT (i.e., 5–80 nM) has been shown to inhibit the GR/GER response in a dose dependent manner. (Weger et al., 2012). In a TR-biosensor developed, tg [thyroglobulin (*tg*)*:mCherry*], that contains a 3.2 kb of tg promoter, three dpf chemical exposed-embryos have been shown to be responsive to goitrogenic compounds, such as resorcinol, KClO_4_, methimazole, 6-propyl-2-thiouracil, ethylenethiourea, pyrazole, and phloroglucinol (Jarque et al., 2018), but has yet to be applied to assess whether chemicals classified as eEDCs can activate this signalling pathway.

### 3.4 Other Zebrafish Models for Studying eEDC-Induced Developmental Neurotoxicity

#### 3.4.1 Functional Brain Imaging

To date, the majority of studies on eEDC-related developmental neurotoxicity using zebrafish and rodent models have focused primarily on the early morphological and genetic phenotypes in specific brain regions of the embryonic brain (i.e., changes in anatomy, neural cell morphology and population and gene expression profiles in the HPG axis and/or hippocampus) and on the behavioural consequences in later life. The mechanisms by which EDC-induced molecular/cellular changes during early life may lead to long-lasting behavioural phenotypes, however, remain largely unknown. Real-time responses to eEDCs on the neurophysiology in the embryonic brain could help fill this knowledge gap.

Functional brain imaging offers this possibility. With this in mind, we have established a 4D functional imaging system using a calcium sensor *elavl3:GCaMP6s* TG zebrafish model in combination with light sheet microscopy (LSM) and a GCaMP6s image processing pipeline (Winter et al., 2017; Winter et al., 2021) (listed in Table 1). In this system, a full brain volume readout of neurological activity (*via* calcium images) for zebrafish embryo/larvae can be captured in around 1.8 s. Taken repeatedly over time (over a period of a few minutes) this allows for the identification of region of interest (ROI)-specific neuronal activity data by applying the 3D registration map of four dpf zebrafish embryonic brain. Changes in localised excitability caused by the exposure to neuroactive compounds can be extracted using this system and connectivity maps can then be constructed between such local circuit(s) and other brain regions whose neuronal activity are synchronously tuned by the excitability change in the primary local circuit(s) (Winter et al., 2021). Such functional connectomics in the brain of the zebrafish embryo provides the ability to help translate local eEDC-induced molecular/cellular changes into brain-wide alterations in functional networks, and linking these to complex behavioural phenotypes.

Neural cell type specific TG zebrafish models could further help inform on MoAs underlying eEDC-induced developmental neurotoxicity (Table 1). For instance, TG lines carrying a promoter-reporter system and/or a promoter-GCaMP system for presumptive eEDC-target neuron types would allow for real-time observation of eEDC-induced changes in ontogeny or excitability of that specific neuron type during brain development. Putative candidate neurons for such experiments include GnRH neurons (Palevitch et al., 2009; Wang et al., 2011) and kisspeptin expressing neurons (Song et al., 2015) that are critically involved in HPG axis development and are known to be affected by eEDC exposures in mouse and zebrafish models. TG lines for GABAergic neurons [e.g. tg (dlx6a-1.4kbdlx5a/dlx6a:GFP) (Hoffman et al., 2016)] also offer an important neuron type for examining eEDCs effects as a deficit in GABAergic neurons in the forebrain, particularly in the telencephalon, has been proposed to be associated wih night-time hyperactive behaviour phenotype in an autism model Contactin Associated Protein-like 2 (CNTNAP2) mutant zebrafish (Hoffman et al., 2016). Interestingly, analysis using the behavioural fingerprint database for 550 psychoactive compounds (Rihel et al., 2010) has shown that behavioural characteristics of the *cntnap2* mutant are negatively correlated with those induced by estrogenic compounds (Hoffman et al., 2016). This night-time hyperactive phenotype in the *cntnap2* mutant has been shown to be selectively restored by developmental exposure to phytoestrogen Biochanin A (Hoffman et al., 2016). These data suggest that an estrogen responsive GABAergic pathway in the forebrain may be relevant to autism. Based on some of our recent data that olfactory development is critically regulated by estrogen/EROB cascade (Takesono et al., 2022), examining whether developmental exposure to eEDCs impacts on ontogeny and neurophysiology of a specific neuron composing of olfactory circuits, e.g., OSNs (Miyasaka et al., 2005; Sato et al., 2005), mitral cells (olfactory output neuron) (Miyasaka et al., 2009), inhibitory interneurons (Mack-Bucher et al., 2007), would be a further area of important research.

#### 3.4.2 Combining TG Models

In the natural environment, organisms are exposed to multiple substances with different toxicological potencies and MoAs. Thus, eEDC-related developmental neurotoxicity phenotypes in humans and wildlife will likely result from an integration of effects of multiple toxicants. In a systematic literature search of the human data, four heavy metals (lead, methylmercury, arsenic and manganese) and three eEDCs (PCBs, DDTs and PBDEs) have been listed consistently as “developmental neurotoxicants” (Grandjean and Landrigan, 2014). Given this, assessments on the interactive effects of eEDCs with other neurotoxicological agents on developmental neurotoxicity are warranted. Many neurotoxins, such as heavy metals, induce oxidative stress responses and thus a double TG zebrafish for estrogen biosensor (e.q., *cyp19a1b:GFP*) and an oxidative stress biosensor model would be of great value. A TG zebrafish for detecting oxidative stress *via* the Electrophile Response Element (EpRE) has recently been developed [tg (*EpRE:mCherry*), listed in Table 1 (Mourabit et al., 2019)] and combining this model with the tg (*cyp19a1b:GFP*) model would enable important insights into integrated neurotoxicological effects of, for example, neurotoxic heavy metals and eEDCs on embryonic brain development. Similarly, combining an estrogen biosensor model and with a gene knockout or a genetic mutant of a neurodevelopmental disease model (Table 1) (Miyasaka et al., 2005; Hoffman et al., 2016; Liu et al., 2018) would enable examination of whether a genetic factor predisposition for that neurodevelopmental disease influences the expression of potential biomarkers for eEDC-induced developmental neurotoxicity.

## 4 Concluding Thoughts

EDC exposure during early embryo development has increasingly been recognised as a causative factor of various types of neurodevelopmental abnormalities in animals and humans. However, there is a considerable shortfall in our understanding on molecular mechanisms underlying the EDC-induced developmental neurotoxicity and this has impeded the establishment of a reliable and sensitive biomarker(s) and associated behaviour endpoint (s) for assessing developmental neurotoxicity of EDCs. Consequently, the majority of EDCs remain untested for potential neurodevelopmental effects.

Embryos of TG zebrafish models have considerable potential for application to hazard assessment of eEDCs and other neurotoxic chemicals on embryonic brain development. Biosensor TG zebrafish can provide spatiotemporal information of how eEDCs affect hormone mediated activities in live embryo at various developmental stages, facilitating in the identification of their mechanisms of induced neurotoxicity. Such mechanism-based eEDC-related neurotoxicological information can furthermore be applied into wider contexts related to studies on exposures with other non-EDC neurotoxic agents and/or disease conditions through the use of combined (multiple) TG models including calcium, other biosensors, and/or genetic mutant zebrafish models. These approaches also may furthermore help to identify the most reliable biomarkers for eEDC-induced developmental neurotoxicity that are relevant to a real toxicological contexts animals experience during embryonic brain development.

It is important also to emphasise the need for research to link biomarker analyses for eEDC exposure on neurological development with their effects on behaviour. Although behaviour repertoires of zebrafish embryo/larvae are still limited as compared with those in adult fish, it has been shown that behavioural characteristics in the rest/wake behaviour assay (e.g., night-time hyper activity) in zebrafish embryo/larvae (4–6 dpf) can represent MoAs of neuroactive compounds (Rihel et al., 2010) and behavioural phenotypes in an autism model mutant (Hoffman et al., 2016). Furthermore, we have found that olfaction-mediated avoidance behaviour could be a useful behavioural endpoint for assessing hazardous actions of eEDCs on olfactory development during embryonic brain development (Takesono et al., 2022). We conclude that NAMs based on TG zebrafish embryo-larvae can facilitate in the identification of neurotoxic effects of eEDCs, and other neurotoxic agents, and help to provide the information needed to inform on their associated hazards and in the regulation of their use all of which can be carried implementing the 3Rs principle.

## Glossary

ADHD: attention-deficit hyperactivity disorder
AhR: aryl hydrocarbon receptor
AR: androgen receptor
ASDs: autism spectrum disorders
AVPV: AVPVantero Ventral Peri-Ventricular nucleus
BPA: bisphenol A
BPS: Bisphenol S
CERAPP: collaborative estrogen receptor activity prediction project structure inventory
CNS: central nervous system
CNTNAP2: contactin associated protein-like 2
DDT: dichloro-diphenyl-trichloroethane
dpf: days post fertilisation
E2: estradiol
EDCs: endocrine disrupting chemicals
EE2: ethinylestradiol
eEDCs: estrogenic EDCs
EpRE: electrophile response element
EPA: environmental protection agency
ERE: estrogen responsive element
EROB cells: estrogen-responsive olfactory bulb cells
ERRs: estrogen related receptors
ERs: nuclear estrogen receptors
GABA: gamma-aminobutylic acid
GFP: green fluorescent protein
GnRH: gonadotropin-releasing hormone
GPER: G-protein coupled estrogen receptors
GR: glucocorticoid receptor
GRE: glucocorticoid response element
hpf: hour post fertilisation
HPG axis: hypothalamic-pituitary-gonadal axis
IPCS: international programme on chemical safety
KO: knock-out
LSM: light sheet microscopy
MoAs: the modes of action
MTZ: metronidazole
NAMs: new approach methodologies
NP: nonylphenol
NRs: nuclear receptors
NTR: nitroreductase
OB: olfactory bulb
OECD: the organisation for economic co-operation and development
OSNs: olfactory sensory neurons
PBDEs: polybrominated diethyl ethers
PCBs: polychlorinated biphenyls
ROI: region of interest
RXRs: retinoid X receptors
sEPSCs: spontaneous excitatory postsynaptic currents
SV2b: synaptic vesicle glycoprotein 2b
TBT: tributyltin
TG: transgenic
The 3Rs: replacement, reduction and refinement of the use of animals in research
TRs: thyroid hormone receptors
Vtg1: vitellogenin 1
WHO: the world health organisation
